# Negative pressure wound therapy for extensive soft tissue defects of the lower extremity in a teenager: A case study

**DOI:** 10.1002/ccr3.9188

**Published:** 2024-08-18

**Authors:** Rainer Stanek, Eva‐Maria Pointner, Katja Canigiani de Cerchi, Gerhard Pomberger, Thomas Benkoe, Thomas Hölzenbein, Mehdi Mousavi

**Affiliations:** ^1^ Clinic Donaustadt Department for Pediatric and Adolescent Surgery Vienna Austria; ^2^ Clinic Donaustadt Department for General Surgery, Division Vascular Surgery Vienna Austria; ^3^ Clinic Donaustadt Department of Orthopedics and Trauma‐Surgery Vienna Austria

**Keywords:** children, complex wound, NPWT, pediatric, soft tissue defects, trauma

## Abstract

**Key Clinical Message:**

Negative pressure wound therapy (NPWT) aided in the management of complex soft tissue injury in a 14‐year‐old girl by managing moisture, reducing oedema, and stimulating wound granulation less than 2 months after the accident and prepared the skin for skin grafting, thus successfully saving the limb.

**Abstract:**

This case study describes the use of a tubular bandage encompassing the whole limb to deliver successful NPWT in the treatment and reconstruction of extensive soft tissue damage extending from the right lower extremity to the hip in a 14‐year‐old female.

## 1 INTRODUCTION

Worldwide, road traffic injuries are the leading cause of death for children and young adults aged 5–29 years. Even greater numbers suffer nonfatal injuries and incur disabilities as a result.^1^ In children with serious trauma, the force of the traumatic impact can be transmitted through the child's body resulting in multisystemic damage.^2^, ^3^ If major vascular structures are involved, or if the continuous blood loss leads to haemodynamic instability, soft tissue wounds can be life‐threatening.^4^ Achieving optimal care of complicated wounds is more demanding in children, due to their special needs for management of anxiety, stress and pain and to the demands placed on the parents.^5^

For adults with traumatic wounds, the use of Negative pressure wound therapy (NPWT) is well established as an adjunct therapy to mitigate the need for and extent of reconstructive surgical intervention.^6^ This approach can decrease the time to wound closure by reducing local oedema, promoting angiogenesis and local blood flow.^7^ No clear guidelines currently exist for the treatment of extensive tissue defects in the pediatric population.^8^ However, there is a growing body of evidence that NPWT is effective for treating children; improving time to healing, decreasing the number of painful interventions, and reducing the extent of surgical reconstruction.^9^, ^10^, ^11^ The capacity of NPWT to stimulate the formation of granulation tissue that creates a wound bed for the skin graft^7^, ^9^ has been linked to decreased morbidity, cost, duration of hospitalization and increased patient comfort.^12^ NPWT has also been used extensively as a fixation of split skin grafts in severe traumatic wounds and is associated with improved graft survival resulting in fewer repeated grafts and graft failure complications in adults^13^ and children.^14^ In a case series of 25 pediatric patients that achieved flap closure or secondary healing with NPWT the authors concluded that benefits significantly outweighed the rare complications.^15^

## 2 CASE HISTORY

A 14‐year‐old female was admitted to our emergency department via air transport 1 h after being thrown from a rolled‐over car in a road traffic accident. The patient presented with a fracture of the of pubis, a traumatic rupture of the external iliac artery and vein at the level of the inguinal ligament, as well as deep soft tissue injuries on to the inguinal ligament.

## 3 METHODS

The cause of patient's pulseless, ischemic right leg was confirmed by an emergency computer tomography scan immediately after admission, showing femoral neck and pelvic ring fractures and tears to major vessels in the area. The vascular surgeon performed an autologous vascular graft of the external iliac artery and vein, and the reperfusion was accomplished within 5 h of the accident. Emergency osteosynthesis of the femoral neck and pelvic ring by orthopedic surgery comprised a screw to the right femoral head, a plate in the area of the acetabulum and an external pelvic fixator. Primary closure of the wounds in the groin area was completed within 8 h of admission. Treatment with an antibiotic (tazobactam) and antifungal (Amphotericin B) was initiated. On the second day post trauma a colostomy was performed and a suprapubic bladder drainage (Cystofix (B. Braun)) system installed.

Despite maximized intensive care treatment, the patient developed systemic inflammatory response syndrome (SIRS), persistent inflammatory and catabolism syndrome (PICS), sepsis and subsequent multiorgan failure. The rhabdolmyolysis following the major soft tissue trauma induced hyperCKemia and myoglobinuria leading to acute kidney injury.

The patient's condition worsened and continuous venous hemofiltration using an additional Cytosorb filter was initiated on the second day after admission alongside treatment with vasopressive agents to manage the renal and cardiovascular organ failure, antibiotics (daptomycin, clindamycin, ceftolozane/tazobactam), and antiviral (ganciclovir) for sepsis. The patient required a complete blood exchange transfusion of more than two liters and heparin continuous perfusion following the vascular graft, which led to significant blood loss.

**FIGURE 1:**
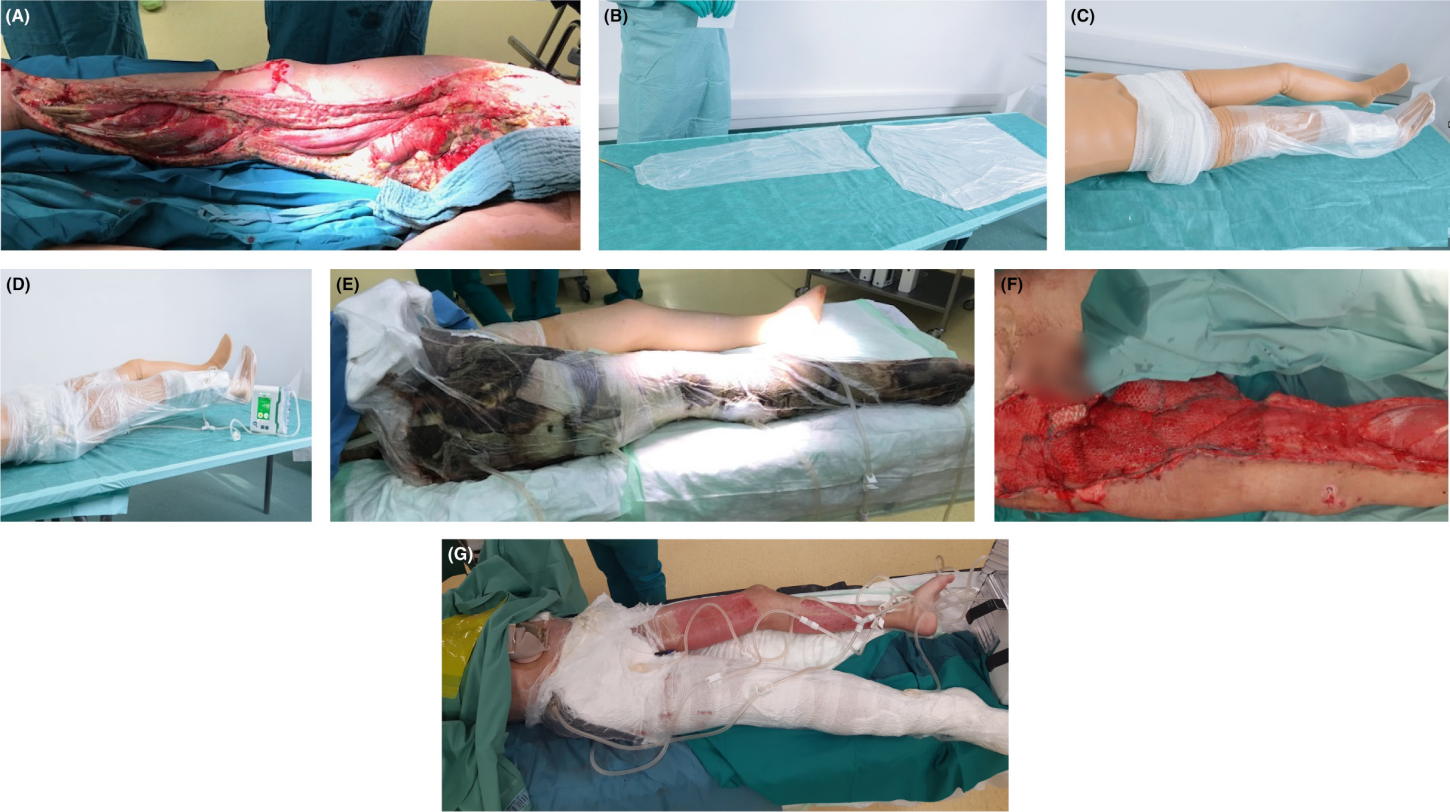
(A) Extensive deep soft tissue defects after large area necrosectomy. (B) Preparation of the Easy Dress L bag for the right leg and Easy Dress fixateur externe bag for the hip area. (C) Coverage of the hip area including fixateur externe with Kerlix and coverage of the right leg with the Easy dress L bag. (D) Coverage of the hip area/pelvis with the Easy Dress fixateur externe bag and leg sealed with self‐adherent foil. (E) NPWT system using a wound foam, PHMB treated gauze, drainage film and nonadhesive tubular dressing. (F) After flap surgery, 2.5 months after accident. (G) NPWT using drainage film, wound foam, and tubular bandage for deeper lesions. NPWT, Negative pressure wound therapy.

Due to the duration of ischemia, the crush nature of the injury, and the reperfusion injury following vascular surgery, the patient developed soft tissue necrosis in the hip and right lower extremity. A fasciotomy for compartment syndrome was performed on the second and fifth day after the accident and the area covered with a synthetic skin replacement (EpiGARD (Biovision)). The pediatric surgery department assumed responsibility for the soft tissue injuries and conducted extensive surgical debridement of necrotic skin, subcutaneous tissue and muscles. Necrosectomy of the right medial femur was performed on the seventh day. Additional necrosectomies to the lower leg and medial femur occurred on days 10 and 14. Figure 1 shows deep tissue defects 8 weeks after the accident. The wounds were colonized with staphylococcus epidermis and pseudomonas aeruginosa. The patient was treated with a broad range of antibiotic, antifungal and antiviral medication.

Given the patient's complex medical situation, and the limited amount of viable tissue available to cover extensive defects in the entire right lower extremity, pelvis and anogenital areas we were forced to consider a series of difficult alternatives as shown below in Table 1.

**TABLE 1:** Details of alternative treatment options considered for patient.

Number of alternatives considered	Details of alternative treatment options
1.	Right thigh‐level amputation
2.	Enucleation of the right hip joint
3.	Amputation of both legs (using the left leg tissue to cover the deep tissue defects)
4.	Hemicorporectomy
5.	Termination of medical treatment and continuing palliative care

In collaborative discussions between pediatric surgery, plastic surgery, vascular surgery, pediatric ICU and neurology, we determined NPWT to be the safest and most promising treatment option available to salvage the limb. The decision was made to commence NPWT 16 days after the patient was admitted (Figure 1B shows preparation of the Easy Dress L bag for the right leg and Easy Dress Fixateur externe bag for the hip area). However, the presence of a colostomy and suprapubic catheter and the fact that the wound extended above the external fixator created significant challenges for ensuring an adequate seal. Our team initially tried to fix NPWT conventionally, using films and gauze cut to fit but could not maintain an airtight seal for longer than 24 h.

After further experimentation we developed a system that allowed us to continue with NPWT (Figure 1C,D for simulation of coverage of the pelvic area with the Easy dress Fixateur externe bag and, the right leg with the Easy dress L bag, secured with self‐adherent foil). The addition of nonadhesive tubular secondary dressings; Suprasorb® CNP EasyDress Fixateur Externe and Suprasorb® CNP EasyDress (Lohmann & Rauscher), which ensured secure fixation, allowed us to maintain a sufficient seal for several days (Figure 1E). This enabled us to set up a schedule in which the NPWT dressing was changed in the operating room every 5–7 days as the patient required a rotating schedule of opioid anesthesia during the procedure. We used the Suprasorb CNP P3 (Lohmann & Rauscher) Therapy System, which delivered negative pressure of −125 mm Hg through three pumps simultaneously, each with two adapter plates. In the initial weeks of NPWT, the device handled exudate volumes of up to three liters per day, and no maceration of intact skin was observed. Amputation at the level of hip joint was to be pursued had the patient failed to respond to NPWT treatment.

We performed wound cleansing with a wound irrigation solution based on NaOCl/HOCl (LAVANOX‐Serag, SERAG‐WIESSNER)”), sponges and monofilament fiber debridement pads (Debrisoft® Lohmann & Rauscher), and sharp surgical debridement with a scalpel. Antimicrobial PHMB impregnated gauze dressings (Kerlix™Cardinal Health) were used to cover intact skin on all areas of the right extremity including the anogenital area up to the iliac crest. A hydrocolloid dressing (Suprasorb® H [Lohmann & Rauscher]) was used on superficial lesions and areas irritated by frequent dressing changes. Double‐sided protective drainage film (Suprasorb® CNP [Lohmann Rauscher]) was wrapped around cut‐to‐fit lengths of black NPWT wound foam and fixed to it with a surgical stapler. These lengths were then placed in the deep extensive defects which generated high volumes of exudate. Large quantities of adhesive film on a roll were used to achieve airtight seals. The external pelvic fixator was wrapped in Kerlix antimicrobial gauze (PHMB) to prevent it puncturing the Suprasorb CNP EasyDress during mobilization. Suprasorb CNP EasyDress Fixateur Externe was adapted to cover the pelvis and fixator by cutting off two corners to provide leg holes and Suprasorb CNP EasyDress was used to cover the leg. A urostomy was carried out and the outlet tube was passed through a cut in the Suprasorb CNP EasyDress which was sealed with a double sandwich of adhesive film. Each dressing change in the operating theater R took an hour and a half to 2 h. With an additional hour and a half for surgical interventions, each procedure lasted approximately three to 4 h and involved multiple personnel. With this system it was possible to control bacterial colonization of the wound, and the patient's haemodynamic condition improved significantly 2 weeks after implementing NPWT.

**FIGURE 2:**
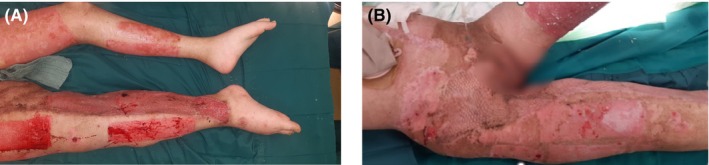
(A) Good graft take‐rate, 3.5 months after the accident. (B) Wound closure 6 months after the accident.

Negative pressure wound therapy was continued until there was sufficient granulation tissue to permit grafting. Two and a half months after admission the patient underwent a flap surgery over the vascular graft in the right groin area (Figure 1F). Harvesting was carried out from the ipsilateral and contralateral lower extremity and 10% of the body surface including the right groin, right calf, right medial, and dorsal femoral region was covered with split skin 6 weeks after initiating treatment with NPWT, Due to the significant psychological implications of her experience, we intentionally selected lower body sites to preserve the patient's intact upper torso and head. The mesh grafts were fixed with fibrin glue and covered with a silicone spacer, Kerlix, CNP therapy, and Suprasorb CNP EasyDress to enable the continuation of NPWT. (Figure 1G). The donor sites were covered with Suprasorb CNP EasyDress and Kerlix. We achieved a good graft take‐rate, despite microbial colonization with 3x Multi‐resistant Gram‐negative (MRGN) Pseudomonas aeruginosa (Figure 2A), three and a half months after the accident.

**FIGURE 3:**
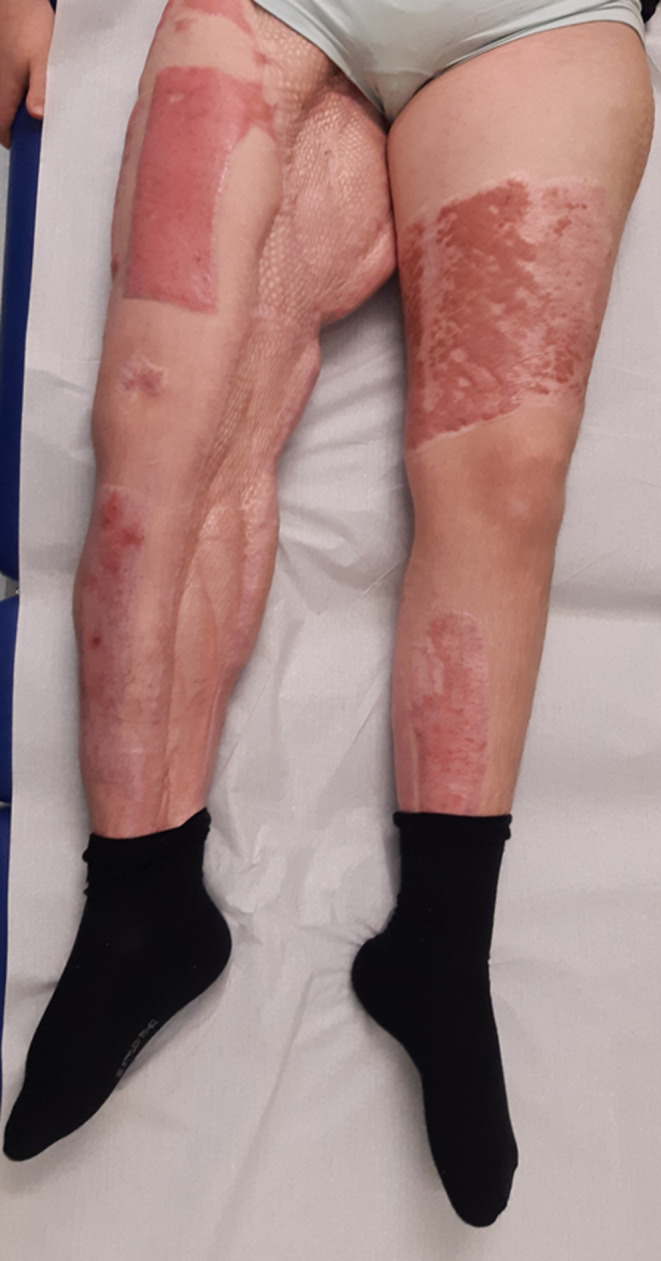
The extremity could be preserved with a good functional result 16 months after the accident.

In total, NPWT was used over 4 months with a total of 30 dressing changes. As the Suprasorb CNP EasyDress allowed the limb to remain mobile, the physiotherapist was able to start mobilization with NPWT in place. The patient was discharged from ICU less than 5 months after the accident. Her deep wounds were completely closed 4 months after starting treatment with NPWT. She achieved full wound closure 6 months (Figure 2B) after the accident but continued to be an inpatient for 10 months for rehabilitation. The patient is now able to walk on the leg (Figure 3). Further orthopedic corrective and reconstructive surgeries are being considered.

## 4 DISCUSSION

The care of pediatric trauma wounds requires a knowledgeable multidisciplinary team to manage prompt assessment, pain control, cleansing, debridement, application of appropriate dressings, and close follow‐up.^4^ Our patient sustained extensive soft tissue defects to the lower extremity with deep substance defects in the hip. Due to their location, extent and level of exudate, such wounds are difficult to treat with conventional dressings. NPWT has shown to be the best option as an adjunct to surgical reconstruction for the treatment of trauma‐related wounds to extremities^16^ and perineum^17^ without significant complications.

In children, advantages of NPWT over other kinds of dressing for complicated wounds associated with tissue loss include less pain, quicker recovery, less frequent dressing changes, possible recovery of exposed surgical hardware, granulation and shrinkage of the wound.^10^ Nonetheless, applying NPWT systems to anatomically challenging locations remains problematic as these areas often have irregular surfaces and an unsuitable environment that interfere with the ability to manage the packing and adhesive dressing effectively enough to create an airtight seal. Resultant air leaks may increase the frequency and duration of dressing changes; delaying healing and increasing costs.^18^, ^19^ Various methods of improving dressing adherence to create an effective seal have been described including: tissue adhesives^18^ hydrocolloid dressings,^20^ silicone,^21^ and stoma paste.^22^ However, anecdotal evidence suggests the use of NPWT therapy in extremely challenging areas is often abandoned.^23^

The establishment of an NPWT system for our patient proved to be difficult and time consuming, with the added complications of navigating an external fixation on the pelvic ring as well as the Cystofix and colostomy. A key factor in her eventual recovery was the introduction of the EasyDress Fixateur Externe which allowed us to continue pursuing NPWT as a viable option.

The usability, quality, and user satisfaction of tubular dressings for NPWT for anatomically difficult to manage wounds have previously been reported. Weinzierl et al. (2019)^24^ used a polyurethane sleeve to cover a forearm with keloid scar excision extending over the elbow joint. In this case report, sealing gauzes or foams on a whole extremity by overlaying a sleeve simplified the dressing process, lowered the total number of manipulations and speeded up execution time. Kolmorgen (2017)^23^ also showed how the tubular sleeve can facilitate the use of NPWT in other difficult anatomical locations such as foot ulcers. These reports support Duft et al.'s (2015)^25^ multicenter observational study that showed how the sleeves saved time, reduced the number of dressing changes and increased comfort for the wearer; attributes that are particularly relevant to our patient as her unstable condition meant the need to complete each dressing change in the Operating Theater was placing additional strain on her body.

In this case, the main advantage of using the EasyDress nonadhesive secondary dressing, was its single point of closure that facilitated an airtight seal to the entire wound including the large pelvic defects. This allowed us to extend the intervals between dressing changes and reduce the total number of interventions requiring repeated anesthesia.

While serious complications are rare in pediatric NPWT patients' dermatitis or maceration of intact skin can be a concern.^26^ Management may involve application of barrier protective gels or barrier dressings to the skin. For our patient, no maceration was noted which we attributed to the high MVTR of the elastic bandage.

Our patient's sepsis improved immediately after the commencement of NPWT. Stannard et al.'s (2009)^27^ RCT evaluating the impact of NPWT after severe open fractures on deep infection found a statistically significant difference in infections (*p* = 0.024) between NPWT patients and the control group, with the relative risk ratio of 0.199 [95% CI: 0.045–0.874] suggesting that the NPWT patients were only one‐fifth as likely to have an infection. Incisional NPWT has also been shown to significantly reduce the risk of SSI after groin incisions for arterial surgery.^28^

While optimal timelines for the rehabilitation of children in intensive care units have not been established, a shift toward early mobilization is gaining momentum in the literature.^29^ With this dressing protocol, our patient's extremity was protected and mobile allowing her to undergo physical therapy simultaneously with NPWT.

The patient experienced highly successful outcomes of her skin grafting surgeries. This was likely positively impacted by the use of NPWT in the weeks prior to surgery. NPWT has been shown to increase not only the quantity of granulation tissue but also the quality. Hence, the graft bed is optimally prepared to accept a full thickness graft.^30^ Compared with historical controls, patients with split thickness skin grafts secured with NPWT need fewer grafting procedures, have very high initial graft survival, and good long‐term wound closure rates.^31^

## 5 CONCLUSION AND RESULTS

Our report describes the care of a child with multiple deep substance defects including loss of cutis, subcutis, and deep muscle defects of gluteus maximus and minimus, multiple organ failure, sepsis, ruptured external iliac artery, and iliac vein. The patient was managed with necrosectomy, osteosynthesis, fasciotomy, serial split skin coverage, flap surgery, and long‐term NPWT. While the safety and benefits of NPWT have been clearly demonstrated in the adult population, studies evaluating its use in pediatrics remain limited.^8^ In this case, NPWT aided in the management of complex soft tissue injury in a 14‐year‐old by managing moisture, reducing oedema, and stimulating wound granulation less than 2 months after the accident, thus preparing the wound bed for a successful skin graft. It also decreased the frequency and complexity of dressing changes, improved comfort for the patient and enabled her to mobilize while wearing the NPWT system.

With a team‐based approach to appropriate treatment options including NPWT, we managed to preserve the patient's limb thereby averting the need for more invasive options such as complete amputation of leg, enucleation of the hip joint, or thigh level amputation. The patient's wounds healed 6 months after the accident. She is now able to walk freely after a secondary orthopedic operation and the lumbosacral defects have healed under conservative treatment.

There is a need to develop clear and concise guidelines and optimal treatment strategies for children and adolescents with complicated tissue defects. Our patient outcomes support the use of an elastic bandage to achieve an airtight seal in NPWT of complex wounds in challenging anatomical locations while navigating invasive medical devices. This report adds to the growing body of evidence for the effective management of large, traumatic wounds in children with NPWT.

## AUTHOR CONTRIBUTIONS

**Rainer Stanek:** Writing – original draft; writing – review and editing. **Mehdi Mousavi:** Writing – review and editing. **Thomas Hölzenbein:** Validation; writing – review and editing. **Katja Canigiani de Cerchi:** Data curation; investigation; methodology. **Eva‐Maria Pointner:** Data curation; investigation. **Thomas Benkoe:** Writing – review and editing. **Gerhard Pomberger:** Validation; writing – review and editing.

## FUNDING INFORMATION

None.

## CONFLICT OF INTEREST STATEMENT

The authors have no conflicts of interest to declare.

## CONSENT

Written informed consent was obtained from the patient to publish this report in accordance with the journal's patient consent policy.

## Data Availability

The data that support the findings of this study are available on request from the corresponding author. The data are not publicly available due to privacy or ethical restrictions.
